# The Human Hair

**Published:** 1859-01

**Authors:** 


### THE HUMAN HAIR.

Baldness is considered a great calamity by many. It is brought on in many cases by wearing the hat too constantly. or by any other means which keeps the head too warm. Another cause of baldness is, the filthy practice of keeping the hair soaked in various kinds of grease, or allowing the scalp to remain unwashed for weeks and months together. Instead of throwing money away for any of the thousand inert, if not hurtful "hair restoratives" which meet the eye in every paper, our readers would do well to at least try the following wash: Pour three pints of hot water on four handfuls of the stems and leaves of the garden "box," boil it for fifteen minutes in a closed vessel, then pour it in an earthen jar, and let it stand ten hours; next strain the liquid and add three table spoons of cologne water; wash the head with this every morning, it is cleansing and tonic, and if the root bulbs of the hair are not destroyed (which is the case where the scalp looks smooth and shiny, and then there is no remedy,) the hair will begin to grow with vigor. If this wash fails after a few weeks perseverance, the baldness may be considered incurable, because the structure of hair growth is destroyed, the cogs and wheels are gone, and no power can replace them, short of that which made them first.

But a more certain and more easily understood method of restoring the hair, when such a thing is possible, is to strive to secure a larger share of general health; keeping the scalp clean in the meanwhile, by the judicious application of a moderately stiff brush, and a basin of plain old-fashioned soapsuds; for, as a general rule, baldness arises from one of three things—inattention, which brought on a decline of health dirt, or stupidity. What for example could a woman expect better than an unsightly broad path of skull along the line where the hair is parted in front, when she has kept each particular hair on a constant strain at the root, at the same identical spot, from earliest " teens" to thirty, instead of changing the line slightly every month or two, or giving entire rest, by having no parting at all, but to carry the hair backward for a month or two at a time, or adjust it in any way which a correct taste and sense of appropriateness will readily suggest to a quick witted woman. In this way the delicate line of parting may be made to look rich and young to the confines of old age.

The judicious cultivation of the hair, that natural ornament, of which when possessed in its abundance, richness and beauty, all are pardonably proud, is most unaccountably neglected; for we are all conscious of the tact, that if the hair is plentiful and is handled with a pure taste, it will add to the impressiveness of any set of features.

As it is, the hair begins to fall before our girls are out of their " teens." In a room full of them, not one in a half dozen can boast of anything on the "back head" but a knot about the size of a hickory nut. If appearances are to the contrary, it will be found that it is a borrowed ornament, whose original owner is in the grave, or has parted with it for a few pennies, or glazy ribbon or gaudy handkerchief, to "raise another crop" just as rich and beautiful. The girls of Brittany and the lower Pyrenees, repair to the annual Hair Fairs in droves, where each one waits her turn for shearing, with her rich long hair combed out and hanging down to the waist. The most valued head of hair brings five dollars, and down to twenty cents, according to quantity and quality. One dollar, in fiery ribbons, violent colored calicos, and the like, is the average, bringing double these prices when taken to the Paris and London wholesale dealers. The weight of a marketable head of hair when first taken from the head is from twelve to sixteen ounces, or from three-quarters of a pound to a pound, under twelve not being " accepted," and over a pound, or sixteen ounces, especially if silken and long, bringing fabulous prices. Rare qualities have been sold at double the price of silver, weight for weight. Two hundred thousand pounds of hair are shorn from the heads of young girls every year, to supply the demands of the Paris and London markets, and from these we derive our supplies.

The hair "growers" seem to be rather a degraded set of people, living in mud huts, in filthy community, garments so patched and worn as to scarcely hold together by their own weight. For once at least, fashion bows to profit, and the richest and most luxuriant head of black hair is accounted an incumbrance. Caps are worn by these people, so as to conceal the hair almost entirely. So, as far as personal appearance is concerned, it would seem of very little consequence whether they had any hair or not. But an important practical hint may be taken from this historical fact. Caps being thus worn there is no need for combs and pins and plaits and ties, and as a consequence no hair is strained at its root, nor is it distorted by being pulled against the grain—against its natural direction.

The Manillans have the longest, blackest and most glossy hair in the world. They do not wear caps at all, but allow the hair to fall back behind in its own natural looseness. Taking these two facts together, it would seem that one condition for having a fine head of hair is, that it should never be on a strain, and should hang pretty much in the direction of its growth, or if diverted at all, as from over the face, it should be in a gentle curve over and behind the ears, with a loose ribbon to keep it from spreading too much at the back of the neck, the hair hanging its length down the back.

The girls of Brittany wear their hair under their caps, so as to conceal it entirely, and those of Manilla having theirs still longer, more glossy and abundant, wear no caps at all but allow it to fall loose over the shoulders. One instructive circumstance connected with this richness of female ornament is, that in both, one condition is present; the hair is not strained against its natural direction, nor indeed is it strained at all. But there is one other condition in the case of the Manillans, which may aid in causing that superiority in length, glossiness, and abundance—it is not braided or tied, or knotted up in any way, but floating in perfect freedom—a thorough ventilation is allowed. It has been found by observant ladies, that when nature is aided in respect to ventilation, by redding the hair very gently and freely night and morning with a fine tooth comb, its richness, glossiness, silkiness, and length, are all increased, as the following incident, related by a traveller strikingly illustrates. He stated that he fell in with a man, whose bearing indicated that he was a gentleman, one of position, and of unusual scholastic attainments; but without these, there was a singularity about him which would have forcibly arrested the attention of the most careless observer; his hair was the longest, most abundant, the most silkenly beautiful that he had ever observed in man or woman either, and more, he seemed to bestow a large share of his attention upon it, and he was evidently proud of it. He spent a great part of his time, when not necessarily engaged otherwise, in combing it, exhibiting in the operation a carefulness, a delicate and gentle tenderness, amounting almost to an affection. At night, he bound it up, so as not to be strained or tangled in any manner. Our traveller's curiosity was excited, and he rested not, until he learned that the gentleman in question was a minister of some religious sect, and that his order was debarred every personal adornment, except that of the hair, which was allowed to be cultivated and worn to any desired extent. The priest gave as his opinion that the success of his cultivation depended on gently combing it a good deal in the direction in which it grew, and preventing all strain beyond that of its own weight.

This mode of treating the hair is strikingly opposed to that prevalent among us, the practice being to begin, in almost infancy, to part the hair in front, and plait it, and knot it, and strain it, almost to pulling it out sideways, crossways and upwards; the ingenuity being taxed apparently to strain it in every direction, so it be contrary to that which it would naturally take; not only so, but the meanwhile it is kept saturated with any and every kind of grease, tallow, hog's fat and rancid butter, disguised, intermixed, or partially purified, and then with a flourish of trumpets and certificates, written by knavery, signed by stupidity, and published abroad unblushingly to the end, that while the fabricators and falsifiers make money, our daughters' heads become mangy, the hair dropping out, the scalp becoming diseased, giving head aches, dullness, smarting eyes and a dozen other correlative symptoms. Then comes a subterfuge and a degradation both together, in order to make up for the deficiency, and some dead corpse is robbed, or some filthy Breton or Manillan is despoiled, the deception not being known until the marriage ceremony has made it too late to be remedied. Out upon it we say, these shams of ivory, and cotton batting and hair of people dirty or dead. Why, most of us young men, if we marry at all, have to risk marrying parts of half-a-dozen people at once.

The lessons learned by these statements are—

1. The hair of children should never be plaited, or braided, or twisted, or knotted.

2. Nothing should ever be put on it except simple pure water, and even this not until the scalp is cleaned.

3. The hair should be kept short. It would be a valuable accomplishment, if when a woman becomes a mother, a few lessons were taken from a good barber, so that the child's hair after the third year, might be trimmed by its mother once a week, only cutting off the longest hairs, by ever so little, so as to keep it of a uniform length. This practice is proper for male and female, old and young.

4. The hair should be always combed leisurely and for some considerable time, at least every morning, and neither brush or comb ought to be allowed to pass against the direction of the hair growth.

Pomatums and hair oils, and washes of every description are wholly pernicious and essentially disgusting, because they detain on the hair and scalp that dust and those animal excretions, which otherwise would fall off or be blown away. The most perfect cleanliness of the scalp, should be sedulously labored for, the first step being that of pure soft water (rained or distilled,) applied by rubbing it in upon the scalp, with the "balls" of the fingers, thus avoiding wetting the whole mass of hair when long; after it is thoroughly dried, then it should be patiently followed by a brushing in its dry state, in the direction of its growth. This is most assuredly the best way to give the hair all that beauty and polish of which it is susceptible. It is abundantly soon to allow the hair of girls to begin to grow long, on entering their fourteenth year, nor should it be allowed to be parted in front sooner than two or three years later, if there be any desire to have the "parting" delicate, beautiful and rich. But all this while, there should be secured the same perfect cleanliness of scalp; the same daily ventilation at the roots; the same daily redding and brushing in its dry state, it being done leisurely and long; while the clipping should be made every fortnight, but only of those hairs which have outgrown the others, or which may have "split" at their ends. Do not "thin" the hair, only cut off the smallest length of the straggling or most lengthy; the object being a greater uniformity as to length, preventing thereby any undue or irregular straining in handling.

As the hair of most persons tends to curl in some direction, that direction should be noticed and cultivated, when a beautiful curling is desired.

As a general rule we would discourage any application to the hair, but if on some rare occasion, we desire to give greater firmness or durability to any particular adjustment of it, in curling or otherwise, a very weak solution of isinglass is the best thing that can be employed.

And if at times any "falling off" is observed, and it is desirable to arrest it sooner than mere cleanliness, and improved health would do it, one of the most accessible washes, is boiling water poured on tea leaves, which have already been used and allowed to stand twelve hours, then put in a bottle and used as a wash to the scalp, it should be of moderate strength. Another good wash is one grain of spirits of tannin, and six ounces of spirits of Castile soap, well rubbed in the head every morning, a table spoon or two at a time, until the hair ceases to fall off.

Curling tongs and papers are destructive to the hair. If any thing is used on an uncommon occasion, it should be silk, or the very softest paper as near the color of the hair as possible. The hair should not be tied at any time with a string, but loosely with a thin soft ribbon, or carried in a loose twist on the part of the neck about the line of the hair, so as to avoid all straining, especially against the direction of the hair growth. The almost universal custom of our women of drawing it up from behind, for the purpose of wearing it at the back of the head, or at the top, is contrary to good taste and physiological wisdom, the great point being to wear the hair without any strain upon its roots beyond its own weight, and loosely, so as to afford a constant, free, and thorough ventilation. It is a great mistake that water "rots" the hair; it is accumulated dust and dirt and grease which does that. Water lightly applied to these accumulations, becomes hurtful by merely softening them, but if pure soft water is cleansingly applied, it is in every way beneficial.

